# Pouch failures following restorative proctocolectomy in ulcerative colitis

**DOI:** 10.1007/s00384-020-03680-1

**Published:** 2020-06-26

**Authors:** Ilona Helavirta, Kirsi Lehto, Heini Huhtala, Marja Hyöty, Pekka Collin, Petri Aitola

**Affiliations:** 1 Department of Gastroenterology and Alimentary Tract Surgery, Tampere University Hospital, Teiskontie 35, FI-33521 Tampere, Finland; 2 Faculty of Medicine and Health Technology, Tampere University, P.O. Box 100, FI-33014 Tampere, Finland; 3 Faculty of Social Sciences, Tampere University, FI-33014 Tampere, Finland

**Keywords:** Restorative proctocolectomy, Ileal pouch, anal anastomosis, IBD surgery, Pouch failure

## Abstract

**Purpose:**

Restorative proctocolectomy (RPC) is the most common operation in ulcerative colitis. Nevertheless, permanent ileostomy will sometimes be unavoidable. The aim was to evaluate the reasons for pouch failure and early morbidity after pouch excision.

**Methods:**

The number and the reasons for pouch failures were analysed in patients undergoing RPC 1985-2016.

**Results:**

Out of 491 RPC patients, 53 experienced pouch failure (10 women, 43 men); 52 out of 53 underwent pouch excision. The cumulative risk for excision at 5, 10 and 20 years was 5.6, 9.4 and 15.5%, respectively. The reasons for failure included septic events such as fistula in 12 (23%), chronic pouchitis in 11 (21%) and leakage in 8 (15%) patients. Functional reasons for pouch failure were recorded as poor function in 16 (30%), incontinence in 12 (23%) and stricture in 12 (23%) patients. Multiple causes for pouch failure were recorded for individual patients. Seven cases of Crohn’s disease were found among the failure cases: two before pouch excision and five after. Altogether, 15 Crohn’s disease diagnoses were set in the RPC cohort, giving a percentage of 47% of pouch failure in this disorder. A complication occurred in 23 (44%) patients within 30 days after surgery; 16 were mild (Clavien-Dindo grades I–II).

**Conclusions:**

Eleven percent of RPC patients suffered pouch failure: more men than women. The reasons were multiple. Crohn’s disease created a risk of failure, but a half of these patients maintained the pouch. Morbidity after pouch excision was moderate, but in most cases slight.

## Introduction

Since its introduction by Parks and Nicholls in 1978 [1], restorative proctocolectomy has become the procedure of choice in patients with ulcerative colitis (UC) requiring surgery. Despite the evolved details of the operation and centralization of the surgery to centres having the number of operations on an acceptable level, poor functional results and pouch-related complications may compel excision of the pouch and the construction of a permanent stoma. The long-term failure rate for RPC is reported to be 10–15% [2–4]. The most common causes of pouch failure have been pelvic sepsis, poor pouch function, pouchitis and Crohn’s disease [4–6]. The factors leading to pouch excision are still poorly understood. This prompted us to explore the long-term risk of pouch failure, covering more than three decades of RPC cases in a high-volume hospital. We report the reasons for pouch failure, factors associated with the risk and the morbidity of pouch excision.

## Material and methods

All consecutive patients with UC undergoing RPC at Tampere University Hospital between 1985 and 2016 were identified from the patient records using the ICD-9 and ICD-10 codes for UC and NCSP (Nordic Classification for Surgical Procedures) codes for the types of operations. A database to form an RPC registry was collected from patient files including details on patient history, surgical technique, postoperative morbidity and follow-up. On patients with pouch failure, we collected details on patient history, reasons for failure, treatment prior to failure and on the pouch excision procedure. Seventy-five patients had moved elsewhere in Finland. Data on the pouch excision of these patients were retrieved from the register of the National Institute of Health and Welfare (NIHW). The patients were identified by the NSCP procedure codes. A widely used prognostic model, the Charlson Comorbidity Index, was used to categorise pouch failure patients’ comorbidities, a method based on the diagnostic codes [7]. Each comorbidity category had an associated weight (from 1 to 6, 6 being the most severe) based on the adjusted risk of mortality or resource use, and the sum of all the weights yields a single comorbidity score for a patient. A score of zero indicates that no comorbidities were found. The higher the score, the more likely the outcome to result in mortality or higher resource use. Complications were classified according to the Clavien-Dindo (C-D) classification, consisting of seven grades (I, II, IIIa, IIIb, IVa, IVb and V) [8]. Crohn’s disease was diagnosed in endoscopy, either by pouch endoscopy or endoscopy from stomal entrance, histology, capsule imaging or magnetic resonance imaging (MRI) and clinical picture.

All pouches in our RPC registry are of J-type and the anastomosis is either handsewn or stapled. Pouch failure was defined as the need for a permanent ileostomy with or without pouch excision. Early complications were defined as occurring within 30 days of the operation.

## Statistical analysis

The data were analysed using SPSS (Version 25.0 IBM SPSS Statistics for Windows, Version 25.0. Armonk, NY: IBM Corp.). For categorical variables, the results were given as frequencies and percentages, and for continuous variables as means and standard deviations or as medians. Chi-square or Fisher’s exact test was used to assess differences in categorical variables. The binary logistic regression analysis was used to determine the predictors of pouch failure*.* Results are shown as odds ratios (ORs) with their 95% confidence intervals (CIs). The cumulative probability of pouch excision was estimated by the Kaplan-Meier method. Statistical significance was set at *p* ≤ 0.05.

## Results

### Basic

The study group comprised 491 RPC patients: 209 women and 282 men. The median follow-up time of the RPC cohort was 11 years (0–33). The data was gathered and analysed during 2017. The baseline characteristics of the patients in the RPC cohort and the pouch failure cohort are presented in Table 1. All the pouches were J-pouches and only five pouch operations were laparoscopic. Thirteen (25%) patients were smokers at the time of pouch excision. This information was gathered from anaesthesia forms, and data were not available in two cases. The Charlson Comorbidity Index distribution among pouch failure patients was 0 in 89%, 1 in 7.5% and 2 in 4% of the cases.

**Table 1:** Demographic, ileal pouch-anal anastomosis (IPAA), surgery related and information of the J-pouch and pouch failure cohorts

	Pouch failure		Pouch in place		*p*-value
	*n* = 53		*n* = 438		
	*n* or median	% or range	*n* or median	% or range	
Men	43	81	239	55	< 0.001
BMI at time of IPAA, mean, (SD)	25	(4.7)	25	(3.4)	
Age at time of IPAA (years), median (range)	36	(18–71)	37	(18–72)	
Anastomoses					
Handsewn	45	85	243	56	< 0.001
Stapled	8	15	195	45	
Covering stoma	19	36	250	57	0.003
Hospital stay (days), median (range)	9	(4–42)	11	(2–48)	

### Failure

**Fig. 1:**
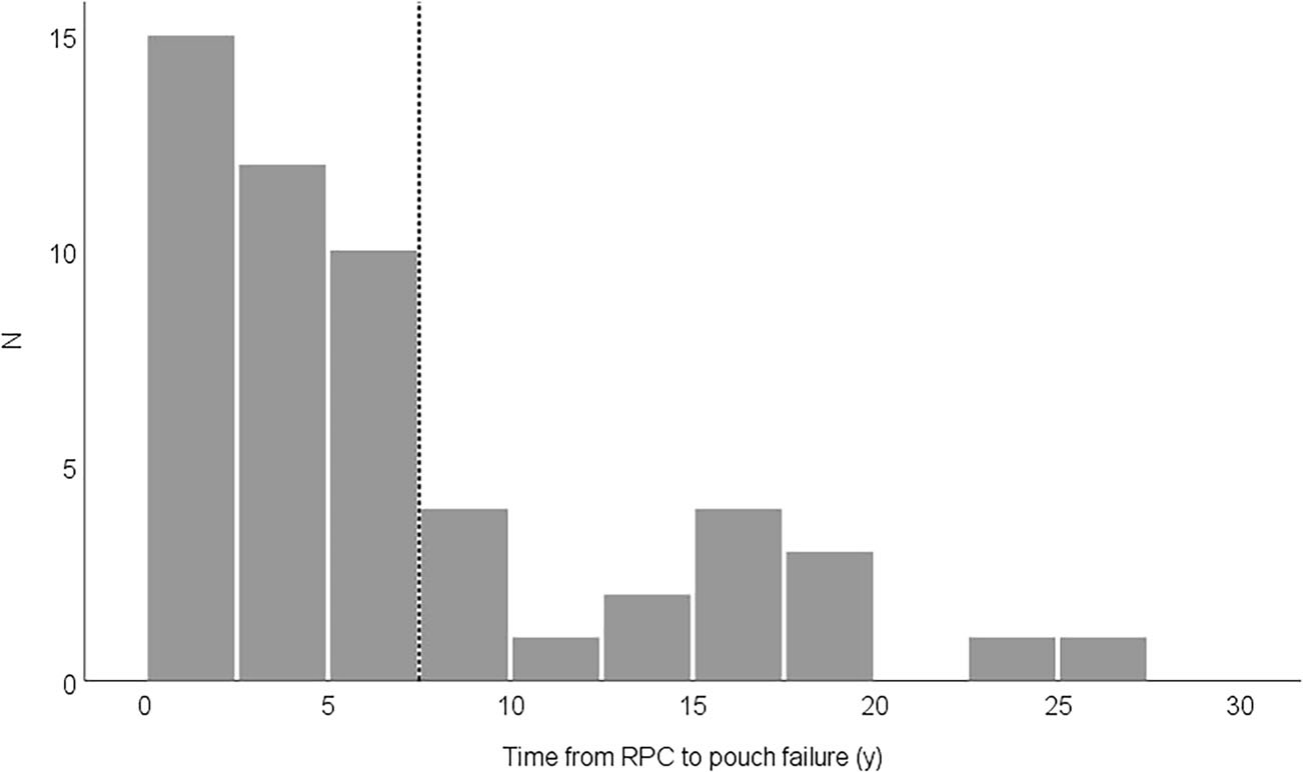
Time (years) from RPC to pouch excision. The majority of pouch failures occurred within 7 years of RPC

Altogether 53 (10.8%) pouch failures were recorded: in 10 (4.7 %) out of 209 women and in 43 (15.2 %) out of 282 men OR 3.58 (95% CI 1.75–7.37). Of these, 52 underwent pouch excision and one permanent loop stoma was built due to a leakage. The median time from the operation to the failure was 4.7 (0–26) years, in women 5.0 (0.7–17) and in men 4.7 (0–26) years. Half of the pouch failures occurred within the first 5 years and 70% within 7 years of RPC surgery (Fig. 1). The cumulative risk for pouch excision at 5 years was 5.6%, at 10 years 9.4% and at 20 years 15.5%. Multiple causes for pouch failure were recorded for individual patients as presented in Table 2. Twenty-eight (52.8%) had undergone surgery prior to pouch excision in order to maintain the pouch (Table 3).

**Table 2:** Several reasons for pouch failure recorded for some patients

Reason	*N*	%
Poor function	16	30
Incontinence	12	23
Fistula	12	23
Stricture	12	23
Chronic pouchitis	11	21
Leakage	8	15
Pouchitis	7	13
Crohn’s disease	7	13
Bleeding	1	2
Perforation	1	2

**Table 3:** Prior operations aiming to prevent pouch failure

Operation	*N*	%
Anastomotic dilatation	12	23
Loop stoma	8	15
End stoma	6	11
Fistula operation	6	11
Abscess drainage	3	6
Anastomotic repair	2	4
New ileoanal anastomosis	1	2

### Histologic findings

In pouch endoscopy (carried out in 47 cases) prior to excision, inflammation was seen by inspection in 55%, fistula in the pouch in 4%, fistula in the anastomosis 11%, stricture in the longer limb in 11% and stricture in the anastomosis in 11%. Pouch biopsy histology was available for 42 patients and showed mild inflammation in 29%, chronic active inflammation in 55% and granulation tissue in 2.4% of the patients; in 14% the histology was interpreted to be normal. Of the 48 operation specimens, histology active inflammation was seen in 37 (77%), fistula in three (6.3%), abscess in three (6.3%), stricture in two (4.2%) and mucinotic carcinoma in one (2.1%) and one was interpreted to be normal. In four cases, it was no longer possible to find the pathology statement and one patient died soon after the operation and the specimen was not analysed.

### Crohn’s disease

Altogether, 15 Crohn’s disease diagnoses were set in the RPC cohort after the initial diagnosis of UC; pouch failure occurred in seven (47%) while the frequency in UC was 9.7%. In five of these seven cases, the diagnosis of Crohn’s disease was made after pouch failure. The median age was 47 years (21–72), and 1 (14%) of these patients were female. In those with final diagnosis of UC and pouch failure, the median age was 44 years (26–53), and 8 (17%) were female.A closer inspection of pouch endoscopy and operation histology of these Crohn’s patients revealed two longer limb strictures and pouch strictures while histology showed mainly chronic active inflammation. Postoperative histology showed inflammation in all patients.Of the eight patients with their pouch in place and a diagnosis of Crohn’s disease, four (50%) had died: one died of breast cancer, and the cause of death of the three patients remained obscure. Five of the patients were taking azathioprine and one was taking biologic drugs, and all were satisfied with their pouch function.

### Predisposing factors

Although pouch failure was more common in men than in women, there were no significant gender differences in the causes of pouch failure, medication prior to failure or operations performed prior to failure. In the whole RPC cohort, there was no difference in leakage 15 (7.7%) vs. 31 (11%) or in the occurrence of pelvic sepsis, 32 (15.3%) vs. 43 (15.2%) between women and men.Median BMI at the time of RPC did not differ between those retaining the pouch and those suffering pouch failure 24.2 vs. 25.0%. BMI was drawn from the patients’ anaesthesia forms: in 11 subjects, the information was missing data. In the pouch failure group, the BMI could not be analysed in three cases.None of the five laparoscopically treated patients had experienced pouch failure during their rather short follow-up.We divided the RPC patients into ≥ 65 and younger and there was no difference in pouch failure for these groups 2 (11.8%) vs. 51 (10.8%) *p* = 0.896, respectively.

More early leakage occurred in failure patients than in those with functioning pouch; 15 (28.3%) vs. 32 (7.3%) *p* < 0.001, and also pelvic sepsis, 15 (28.3%) vs. 60 (13.7%) *p* = 0.005, respectively. In the pouch failure group, early relaparotomy was carried out on 11 (20.8%) and of those with functioning pouch on 31 (7.1%), *p* = 0.003. Covering stoma had been used in 19 (35.8%) in the pouch failure group and 250 (57.2%) patients in the pouch in place group, *p* = 0.003. The anastomoses were handsewn in 45 (84.9%) and in 243 (55.5%) patients, *p* = <0.001, respectively. The univariate and multivariate analyses of the demographic, surgery related and complication data are presented in Table 4. The only significant parameters in the multivariate analyses were leakage, OR 3.65 (95%CI 1.21–1.83) *p* = 0.022 and male gender OR 3.83 (95%CI 1.80–8.12) *p* ≤ 0.001.

**Table 4:** Univariate and multivariate analyses of demographic, surgery related and RPC complication information on RPC and pouch failure cohorts

	Univariate			Multivariable		
	OR	95% CI	*p*-value	OR	95% CI	*p*-value
Men	3.58	1.75–7.31	< 0.001	3.83	1.80–8.12	< 0.001*
BMI at time of RPC, mean, SD	1.01	0.94–1.08	0.847	1.01	0.92–1.12	0.764
Age at time of RPC (years) median (min–max)	1.01	0.99–1.03	0.398	1.01	1.01–0.99	0.419
Anastomoses	4.51	2.08–9.80	< 0.001	2.01	0.66–6.10	0.218
Handsewn						
Stapled						
Covering stoma	2.39	1.32–4.32	0.004	0.87	0.41–1.83	0.218
Pelvic sepsis	2.49	1.29–4.80	0.007			
Leakage	5.01	2.49–10.06	< 0.001	3.65	1.21–1.83	0.022*
Relaparotomy after RPC	3.44	1.61–7.34	0.001	1.21	0.35–4.19	0.764
Year of RPC	0.93	0.90–0.96	< 0.001	0.93	0.88–0.99	0.013*

### Operative outcome

All pouch excision operations were performed as open surgery and median blood loss per operation was 325 mL (50–2300 mL). Median hospital stay after pouch excision was 9 days (2–24). Readmission to hospital was recorded for 13 (25%) of the pouch excision patients.

An early complication was recorded in 23 (44%) of the patients after surgery but 16 (70%) of these were Clavien-Dindo grades I–II. Early complications after pouch excision surgery are shown in Table 5. One patient died soon after pouch excision performed due to arterial bleeding at the pelvic floor; the patient declined blood transfusions for religious reasons. Clavien-Dindo grade 3b complication was the cause of surgery in four (7.6%) patients: one due to obstruction, one due to ureter damage, one stoma revision and one perineal abscess drainage.

**Table 5:** Early postoperative data after pouch excision

Surgical complication	*N*	%
Occlusion	9	17
Perineal wound infection	3	6
Intra-abdominal abscess	3	6
Bladder retention	2	4
Laparotomy wound infection	2	4
Stoma necrosis	1	2
Death	1	2
Bleeding	1	2
Perforation	1	2
Damage to ureter	1	2
Erectile dysfunction	1	2
Urinary infection	1	2
Bleeding from nasogastric tube	1	2
Sepsis	1	2
Clavien-Dindo classification		
0	29	56
1	10	19
2	6	10
3a	2	4
3b	4	8
5	1	2
Antibiotics postoperatively	19	37

## Discussion

This study investigated the frequency and reasons for pouch failure after RPC for UC in the second biggest IBD surgery centre in Finland in a period spanning more than three decades. The overall frequency of pouch failure was 10.8%. In a previous report with a large number of patients and in a meta-analysis, similar pouch failure percentages of 9.7 [5] and 8.6% [9], respectively, were seen with a minimum follow-up time of 5 years. The Charlson Comorbidity Index among pouch failure patients was 0 in 89%, which means that failure patients were otherwise mainly healthy. In a Danish national registry study with a median follow-up of 11.4 years, a 5-year risk of 9.1%, 10-year risk of 12.1% and 20-year risk of 18.2% for pouch failure were found [2].

The reasons leading to pouch failure were often multifactorial: one complication leading to another and subsequently to functional difficulties. The reasons reported in different studies may vary according to how pouch problems are categorised or recorded. The most common reasons for failure in our study were septic events in 21(40%) (fistula 23%, leakage 15%, perforation 2%), pouchitis in 13%, chronic pouchitis in 21%, Crohn’s disease in 13% and functional reasons: poor function was seen in 16 (30%), incontinence in 12 (23%) and stricture in 12 (23%) patients. In a British multicentre study comprising 94 pouch failure patients, the causes were similar: septic complications in 33 (35.1%), poor function in 29 (30.8%), pouchitis in 9 (9.6%) and Crohn’s disease in 5.3% [4]. Septic complications, deterioration of the functional results and Crohn’s disease have also been reported earlier in 1997 [6] and in large studies in this millennium [5]. We have previously reported our functional results for those with pouch in place and 70% of the patients reported good function of the pouch as measured with the Oresland score [10]. Usually before the pouch failure, the function is devastating and the patient is too tired to consider new procedures to save the pouch.

Here, in subjects with pouch failure, the diagnosis of Crohn’s disease was made before in two patients and after failure in five. Altogether, in patients with Crohn’s disease diagnosed after RPC, failure was seen in 47%. This is similar to the findings obtained in large studies [11, 12]. No granulomas were found in our Crohn’s series. A similar result was reported in the Mayo Clinic study with 35 Crohn’s disease pouch failure patients; in only seven cases was pathological confirmation given [13]. In the whole RPC series, the frequency of Crohn’s disease was only 3%. The histologic variability of Crohn’s cannot give an exact diagnosis in every case, but the diagnosis is a combination of the clinical picture, X-ray findings and histology. Nevertheless, in cases with pouch failure, the diagnosis of Crohn’s disease should be borne in mind. In our series there was no significant age or gender difference for pouch failure for Crohn or UC diagnosis.

In our study 15.2% of men and 4.7% of women suffered pouch failure. In earlier studies, there has been no significant difference in gender distribution [14–16] except in the Danish national registry study, which reported a risk in women 39% greater than that in men over time [2]. In early failures, pelvic sepsis due to anastomotic complications played a major role but there was no difference between genders in this report. Nor was there any difference between genders in median time from RPC to failure. We found no explanation for this gender distribution in our study.

Leakage was found to be an independent risk factor for pouch failure OR 3.65 (95%CI 1.21–1.83) *p* = 0.022. This has been reported in several reports before and the prompt diagnose and treatment of leakage cannot be stressed enough [17].

The differences in the uni- and multivariate analyses for the use of covering stoma and type of anastomoses are explained with the changes in practice through the years: handsewn anastomoses were performed mainly in 1985–2005 and covering ileostomy when considered necessary in 1985–2005 and as a routine after that. The year of IPAA has also been taken into account there and it obviously affects the follow-up time.

In our study, all but one of the pouch failure patients (52, 98%) underwent pouch excision. In earlier reports, this percentage has been smaller: in the study by Foley 40% [18], Tulchinsky and Meagher 60% [5, 19] and McRae 84% [20]. In another Finnish study, all 52 failed pouches were also excised [21]. In the present study, 8 (15%) out of 52 patients underwent a loop ileostomy and six patients had an end stoma built before the pouch excision operation. A pouch left in place may burden patients with incontinence. There is a need for guidelines on the management of pouch failure patients; the issues to consider are morbidity of the pouch left in place, morbidity after pouch excision, danger of dysplasia and follow-up of the redundant pouch.

In this study, morbidity of the excision was frequent but most of the complications were mild and thus acceptable. There are very few studies on morbidity after pouch excision. Karoui et al. concluded that early and especially late morbidity is high and pouch excision RPC patients should be initially counselled on this [22]. Prudhomme et al. studied perineal wound healing and divided the patients into groups by diagnosis. Crohn’s disease seemed to be a major risk for perineal wound healing [23]. Bengtsson et al. [24] analysed their diverted pouch failure patients who did not undergo excision (*n* = 22) and found no dysplasia or cancer in histopathology in pouch biopsies; none of the patients requested further surgery and the majority had satisfactory ileostomy function. Altogether, dysplastic transformation in the ileal pouch has been estimated to be rare [25]. The present study supports this view as no dysplasia was found in the postoperative histology. One patient died of mucinotic cancer after pouch excision; this cancer was not found preoperatively despite extensive investigations.

The study was limited due to its retrospective nature and lack of systematic follow-up of the pouch failure patients.

## Conclusions

Although most of the RPC patients operated on for UC had satisfactory functional outcome, 10.8% experienced pouch failure in the long run. The most common reasons for failure were deterioration of functional results after septic complications. Pouch excision is a major surgery and associated with moderate morbidity, necessitating precise planning and patient counselling. The possibility of Crohn’s disease should be considered in UC patients with pouch failure who have been operated on.

## Data Availability

The data is available upon the request from the corresponding author.
